# Editorial: Mind, body, plate: investigating disordered eating in the active population

**DOI:** 10.3389/fnut.2025.1592265

**Published:** 2025-04-29

**Authors:** Gordana Kenđel Jovanović, Renata Barić, Alessandra Pokrajac-Bulian, Zrinka Greblo Jurakić

**Affiliations:** ^1^Department of Environment Protection and Health Ecology, Teaching Institute of Public Health of Primorsko-Goranska County, Rijeka, Croatia; ^2^Department of Health Ecology, Faculty of Medicine, University of Rijeka, Rijeka, Croatia; ^3^Faculty of Kinesiology, University of Zagreb, Zagreb, Croatia; ^4^Faculty of Humanities and Social Sciences, University of Rijeka, Rijeka, Croatia; ^5^Faculty of Croatian Studies, University of Zagreb, Zagreb, Croatia

**Keywords:** eating disorders, athletes, nutrition, psychologic, body image

Athletes face unique challenges, particularly societal expectations, social comparisons, and the pressures of sports performance, which increase their susceptibility to disordered eating. If not recognized and untreated, these behaviors can severely affect athletes' physical and mental health, quality of life, and psychosocial functioning, underscoring the need for professional support (1, 2). Current data on the prevalence of eating disorders, combined with tailored assessment tools for eating behaviors, psychological symptoms, and risk factors in athletic populations, can provide a clearer picture of the prevalence of eating disorders in athletes and support more effective management strategies. Understanding the nutritional needs and body image concerns of athletes is crucial to promoting their physical and mental wellbeing. A narrative review by Amawi, AlKasasbeh et al. highlighted the importance of meeting the specific nutritional demands of athletes and provides evidence-based recommendations to optimize athletes' dietary practices. Dietary choices among athletes are influenced by knowledge, attitudes, and access to nutrition-related resources, making it critical to provide them with access to evidence-based materials. Adolescents, in particular, require greater nutritional support to achieve both their athletic performance and specific growth and development needs. In the context of junior athletes, there is a notable lack of comprehensive, evidence-based guidelines that specifically address their dietary requirements. Amawi, Khataybeh et al. emphasized the critical role of individualized nutrition strategies, including the use of supplements, to support the development and performance, overall health and athletic success of young athletes. The authors highlighted the importance of making dietary choices based on knowledge and attitudes while ensuring access to evidence-based nutritional resources. In addition, the authors emphasized the need for more focused studies to fill this knowledge gap and provide comprehensive nutrition guidelines for young athletes. Body image dissatisfaction is another significant concern faced by athletes. Jakobek et al., in their study, identified key predictors of body image dissatisfaction among kinesiology students, including perfectionist tendencies and a lack of balance between education and employment. They indicated the importance of addressing psychological factors and providing support to improve body image satisfaction among kinesiology students. Understanding these predictors is crucial for developing effective interventions to promote a positive body image, as kinesiology students and future coaches can significantly influence their athletes. Similarly, athletes also experience body image concerns that are influenced by personal risk factors and sport-specific characteristics. Maurin et al. explored the drive for thinness and muscularity in elite young NextGen athletes. Their study identified personal risk factors, such as low self-esteem and perfectionism, and sport-specific characteristics, such as sports requirements, as key contributors to the drive for thinness and muscularity in NextGen athletes. These findings underscore the need for holistic support to address body image challenges and foster healthy attitudes in young athletes. Thus, the authors concluded that tailored interventions and evidence-based guidelines are essential to promote a balanced and healthy body image in this population. Eating disorders are currently an increasing concern among young athletes, emphasizing significant consequences for their physical and mental health. The ERASMUS+ project Sports Community Against Eating Disorders (SCAED), as presented by Kenđel Jovanović and Čulina, revealed that one in five and one in seven young, non-professional European athletes are at risk for eating disorders, depending on the screening tool for eating disorders used. The authors showed the usefulness of a brief screening tool in detecting eating disorder behaviors in young athletes. Their study confirmed the current knowledge that female athletes and those in weight-sensitive sports are particularly vulnerable to the development of eating disorders, making them important subgroups of athletes for intervention. The authors highlighted the need for targeted programs to address these challenges and promote healthy eating behaviors among young athletes, while the deliverables of the SCAED project may help achieve this. Lifestyle interventions have an important role to play in addressing eating disorders not only in athletes but also in the active population. Irandoust et al. evaluated the long-term impact of five different lifestyle change interventions in individuals with eating disorders using machine learning models. They showed that counseling combined with exercise and dietary programs was the most effective approach. The study findings highlight the potential of personalized health strategies to treat eating disorders and improve the overall wellbeing of individuals in the active population. In conclusion, the Research Topic *Mind, body, plate: investigating disordered eating in the active population* underscores the importance of accessible evidence-based nutritional and psychological support for athletes, particularly women and young ones, both elite and non-professional athletes, to promote their optimal performance and wellbeing. Future research should focus on developing comprehensive guidelines and tailored interventions to address the unique nutritional needs and body image challenges of athletes. These efforts are also crucial for coaches, families, and sports medicine professionals who play a key role in the wellbeing of athletes. Furthermore, more research is needed to examine the effectiveness of various lifestyle interventions in preventing and treating eating disorders and promoting healthy eating behaviors in athletes. Understanding the predictors and risk factors associated with body image dissatisfaction and eating disorders may enable the development of successful strategies to support the physical and mental health of athletes (3), especially young athletes, and contribute to their long-term success in both their athletic and personal lives. Finally, the variation in the prevalence of eating disorders among athletes and the lack of definitive conclusions regarding their causes may be due to the lack of sport-specific diagnostic tools (1), thus, the development and validation of tools is crucial to effectively identify eating behaviors, psychological symptoms, and risk factors in different sports populations, which may ultimately help to prevent eating disorders (2).
